# Reasons for COVID-19 Vaccine Hesitancy Among Chinese People Living With HIV/AIDS: Structural Equation Modeling Analysis

**DOI:** 10.2196/33995

**Published:** 2022-06-30

**Authors:** Yan Yao, Ruiyu Chai, Jianzhou Yang, Xiangjun Zhang, Xiaojie Huang, Maohe Yu, Geng-feng Fu, Guanghua Lan, Ying Qiao, Qidi Zhou, Shuyue Li, Junjie Xu

**Affiliations:** 1 Department of Epidemiology and Biostatistics School of Public Health Jilin University Changchun China; 2 Department of Preventive Medicine Changzhi Medical College Changzhi China; 3 Department of Public Health University of Tennessee Knoxville, TN United States; 4 Beijing Youan Hospital Capital Medical University Beijing China; 5 Tianjin Centers for Disease Control and Prevention Tianjin China; 6 Jiangsu Provincial Center for Disease Control and Prevention Nanjing China; 7 Guangxi Center for Disease Prevention and Control Nanning China; 8 The Second Hospital of Huhhot Huhhot China; 9 Department of Emergency Medicine Shenzhen Hospital Peking University Shenzhen China; 10 Changchun Maternity Hospital Changchun China; 11 Clinical Research Academy Peking University Shenzhen Hospital Peking University Shenzhen China

**Keywords:** COVID-19 vaccine, vaccine hesitancy, PLWHA, structural equation modeling

## Abstract

**Background:**

Many countries and organizations recommended people living with HIV/AIDS (PLWHA) receive the COVID-19 vaccine. However, vaccine hesitancy still exists and becomes a barrier for promoting COVID-19 vaccination among PLWHA.

**Objective:**

This study aims to investigate factors that contributed to COVID-19 vaccine hesitancy among PLWHA.

**Methods:**

The study used a multicenter cross-sectional design and an online survey mode. We recruited PLWHA aged 18-65 years from 5 metropolitan cities in China between January 2021 and February 2021. Participants completed an online survey through Golden Data, a widely used encrypted web-based survey platform. Multiple linear regression models were used to assess the background characteristics in relation to COVID-19 vaccine hesitancy, and structural equation modeling was performed to assess the relationships among perceived benefits, perceived risks, self-efficacy, subjective norms, and COVID-19 vaccine hesitancy.

**Results:**

Among 1735 participants, 41.61% (722/1735) reported COVID-19 vaccine hesitancy. Older age, no other vaccinations in the past 3 years, and having chronic disease history were positively associated with COVID-19 vaccine hesitancy. Structural equation modeling revealed a direct relationship of perceived benefits, perceived risks, and subjective norms with self-efficacy and vaccine hesitancy and an indirect relationship of perceived benefits, perceived risks, and subjective norms with vaccine hesitancy. Moreover, self-efficacy toward COVID-19 vaccination was low. PLWHA had concerns of HIV disclosure during COVID-19 vaccination. Family member support could have an impact on COVID-19 vaccination decision-making.

**Conclusions:**

COVID-19 vaccine hesitancy was high among PLWHA in China. To reduce COVID-19 vaccine hesitancy, programs and strategies should be adopted to eliminate the concerns for COVID-19 vaccination, disseminate accurate information on the safety and efficacy of the COVID-19 vaccine, encourage family member support for COVID-19 vaccination, and improve PLWHA’s trust of medical professionals.

## Introduction

The COVID-19 pandemic has become a global health challenge and poses a serious health threat [1]. Compared with the HIV-negative population, people living with HIV/AIDS (PLWHA) with a weakened immune condition or with comorbidities have an increased risk of having poorer outcomes from COVID-19 [2]. Moreover, PLWHA who are immunocompromised are more likely to have a more severe illness and a longer disease course from COVID-19 [3-5]. Some longitudinal studies have reported that PLWHA have higher COVID-19 mortality than the HIV-negative population [6-8]. Therefore, it is critical for PLWHA to receive vaccines to prevent COVID-19. Many countries and organizations recommended PLWHA to receive a COVID-19 vaccine [9-14]. The Joint United Nations Programme on HIV/AIDS has declared that the COVID-19 vaccines authorized by regulators can significantly reduce the risks of severe COVID-19 illness and death and are safe for most people, including PLWHA [11]. The UK Department of Health and US Centers for Disease Control and Prevention released guidance that recommended PLWHA, regardless of CD4 count, should be vaccinated against COVID-19 [12,13]. In March 2021, China launched an updated COVID-19 guideline that also recommended PLWHA receive COVID-19 vaccines [14].

Although the evidence on the side effects and protective efficacy of COVID-19 vaccination in PLWHA is insufficient, some studies have shown that COVID-19 vaccine hesitancy is higher among PLWHA than HIV-negative people [15,16]. For example, a study in British Columbia, Canada, showed that 65.2% of PLWHA reported intending to receive a COVID-19 vaccine recommended and available to them, which was lower than HIV-negative people (79.6%). That is to say, COVID-19 vaccine hesitancy is higher among PLWHA than HIV-negative people [15]. In a cross-sectional study conducted in Beijing, China, the rate of COVID-19 vaccine hesitancy of the PLWHA population was 27.5%, which was higher than HIV-negative people (17.75%) [16]. Hence, vaccine hesitancy exists and becomes a barrier for promoting COVID-19 vaccination among PLWHA. Vaccine hesitancy was defined by the World Health Organization as the delay in acceptance or refusal of vaccination despite the availability of vaccination services [17]. Vaccine hesitancy was listed as one of the top 10 global health threats in 2019 [18].

It is urgently needed to eliminate COVID-19 vaccine hesitancy and improve the coverage rate for PLWHA who might encounter more barriers and have more concerns about COVID-19 vaccination. A recent study reported that vaccine hesitancy was influenced by various factors, such as perceived benefits and risks of a vaccine, perceived safety of a vaccine, confidence in a vaccine, attitudes toward a vaccine, and an individual’s demand [17,19-21]. Perceived vaccine safety was reported as an essential factor that can lead to a vaccination decision [22]. In other words, people who perceive a vaccine as unsafe are more likely to refuse or delay vaccination (vaccine hesitancy) [23]. Perceived risks of a vaccine could also result in vaccine hesitancy [24]. Moreover, a recent French study that investigated COVID-19 vaccine hesitancy in PLWHA indicated a high hesitancy rate of 28.7%, and PLWHA had concerns about serious side effects of COVID-19 vaccination [25]. Another study that investigated vaccine hesitancy among African American PLWHA demonstrated that people trusted some COVID-19 vaccination sources, such as social service and health care providers, more than others [26].

Although previous studies determined some factors that were associated with PLWHA’s vaccine hesitancy, complex relationships among multiple factors might exist but remain unassessed. A structural equation modeling (SEM) approach that provides a flexible framework to analyze multiple variables and takes into consideration relationships among variables could provide a more compelling explanation of COVID-19 vaccine hesitancy. However, there is a lack of research investigating the factors correlated with PLWHA’s vaccine hesitancy through SEM. Therefore, we designed a survey that investigated factors associated with COVID-19 vaccine hesitancy among PLWHA using SEM. Some factors that were reported in the literature were assessed and included in the model, such as perceived benefits, perceived risks, self-efficacy, and subjective norms. The findings of the study aimed to provide valuable evidence for a deep understanding of COVID-19 vaccine hesitancy, therefore contribute to policy making and programming efforts with the goals of addressing vaccine hesitancy and promoting COVID-19 vaccination among PLWHA.

## Methods

### Study Design

The study used a multicenter cross-sectional design and an online survey mode. Recruitment was conducted in 5 large cities from 4 regions of China between January 2021 and February 2021. These cities included 2 in the North (Tianjin and Beijing), 1 in the Northeast (Hohhot), 1 in the East (Nanjing), and 1 in the South (Nanning). To achieve the study objectives, we have set up the following criteria for cities to be qualified and included in this study: (1) must have community-based organizations (CBOs) providing services to PLWHA; (2) each city has a minimum of 3000 reported PLWHA; (3) COVID-19 vaccination was first scaled up in these sites; and (4) there is an adequate supply of vaccines in these sites.

We used 2 methods to calculate the sample size.

The first one was based on the estimation of the rate of COVID-19 vaccine hesitancy among the PLWHA population based on the clustering sample method. According to a cross-sectional study conducted in Beijing, China, the rate of COVID-19 vaccine hesitancy of the PLWHA population was 27.5% [16]. We first estimated the sample size using the following sample size formula from a simple randomized sampling method. The α is the significance level; if α was 0.05, Z_1-ɑ/2_ could be assumed to be 1.96. δ is the allowable error, and was considered to be 0.05. The p, or the estimated COVID hesitancy rate in the PLWHA population, was considered to be 27.5%. Then, we used the design effect (deff) to further calculate the sample size based on a clustering sampling method. The deff was defined as the ratio of the variance, taking into account the clustering sample design and the variance of a simple random sample design with the same number of observations, deff was considered to be 2 based on previous studies [27-29]. Eventually, a sample size of 613 was initially generated based on a clustering sample study design. A minimum sample size of 852 was acquired after taking into consideration the no response rate of participants (20%) and the portion of unacceptable responses (10%). The sample size formula was expressed as:



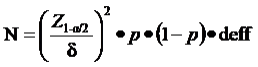



For the second sample size calculation, Nunnally [30] believed that the minimum sample size should be at least 10 times the analyzed variables to conduct a SEM analysis. There are 23 variables in this study without considering background characteristics, so a sample size of 230 was initially needed. A minimum sample size of 320 was acquired after taking into consideration the no response rate of participants (20%) and the portion of unacceptable responses (10%).

Last, we used 852 as the minimum sample size of this study.

### Participants

Eligible participants were individuals aged 18-65 years who had been diagnosed with HIV or AIDS and were living in 1 of the 5 cities. Exclusion criteria included (1) being illiterate and unable to complete the online questionnaire and (2) PLWHA who had been vaccinated against COVID-19.

### Recruitment and Data Collection

We recruited participants mainly through CBOs, which provide services mainly to PLWHA and have been cooperating closely with HIV clinical service providers in the 5 study sites. In China, HIV outreach services to PLWHA have been transferred from government agencies to CBOs [31]. At present, CBOs are the primary providers of these routine tasks. In addition, a large portion of PLWHAs is followed up by CBOs. The questionnaire survey was carried out using Golden Data, a commonly used, encrypted, web-based survey platform. Each participant took about 13-15 minutes to complete this survey. An electronic coupon with a value of 20 Chinese yuan (US $3.10) was sent to the participant upon completion. The database we used was protected by a password and could only be accessed by designated research team members. More detailed information about recruitment and data collection can be found in Multimedia Appendix 1.

### Instrumentation

A panel consisting of public health researchers, psychologists, clinicians, CBO staff, and participant representatives was assembled to develop the questionnaire for the study. Ten participant representatives responded to the online survey and provided feedback for improvement. The panel revised and finalized the questionnaire based on the pilot responses and the feedback. The 10 participants were not included in the final analyses of the study.

The questionnaire included the following sections: (1) background characteristics (eg, including sociodemographic characteristics, presence of chronic conditions, history of other vaccination in the past 3 years, HIV disease information), (2) vaccine hesitancy, (3) perceived risks, (4) self-efficacy, (5) perceived benefits, and (6) subjective norms. Constructs (2) to (6) were assessed using a 5-point Likert scale. Latent variables that may have direct or indirect relationships with COVID-19 vaccine hesitancy were also measured according to the hypotheses.

In this study, vaccination hesitancy was defined as the proportion of respondents who reported “definitely not” or “probably not” or “unsure” to undergo the COVID-19 vaccination program based on a recent peer-reviewed study by Fisher et al [32].

### Study Hypotheses

Based on the literature, we proposed the following study hypotheses:

- Hypothesis 1: Perceived benefit is negatively associated with COVID-19 vaccine hesitancy (the higher the perceived benefits, the lower the degree of COVID-19 vaccine hesitancy).
- Hypothesis 2: Perceived risk is positively associated with COVID-19 vaccine hesitancy (the higher the perceived risks, the higher the degree of COVID-19 vaccine hesitancy).
- Hypothesis 3: Subjective norms are negatively associated with COVID-19 vaccine hesitancy (the higher the subjective norms, the lower the degree of COVID-19 vaccine hesitancy).
- Hypothesis 4: Self-efficacy is negatively associated with COVID-19 vaccine hesitancy (the higher the self-efficacy, the lower the degree of COVID-19 vaccine hesitancy).
- Hypothesis 5: Self-efficacy is positively associated with perceived benefits (the higher the self-efficacy, the higher the degree of perceived benefits).
- Hypothesis 6: Self-efficacy is negatively associated with perceived risks (the higher the self-efficacy, the lower the degree of perceived risks).
- Hypothesis 7: Self-efficacy is positively associated with subjective norms (the higher the self-efficacy, the higher the degree of subjective norms).

### Statistical Analysis

Descriptive statistics were performed to summarize the background characteristics associated with and frequencies of COVID-19 vaccine hesitancy. The total average scores and dimensional average scores for vaccine hesitancy, perceived benefits, perceived risks, self-efficacy, and subjective norms were generated. A 1-way ANOVA test was used to identify the factors predicting COVID-19 vaccine hesitancy. SPSS software (version 24.0; IBM Corporation) was used to perform all data analyses. The significance level was set at a 2-tailed *P* value of <.05.

### Model Analysis

First, means and standard deviations were generated to describe the basic information; skewness and kurtosis were computed to describe the distribution of the data. Furthermore, we used Amos 24.0 to construct the SEM and used the nonparametric percentile bootstrap method of bias correction to test the indirect relationships.

### Ethics Approval

The institutional review boards of the Changzhi Medical College (RT2021003) approved this study. Respondents were informed that their participation was voluntary, and consent was implied by completion of the questionnaire.

## Results

### Background Characteristics

A total of 1883 PLWHA completed the online survey from the 5 metropolitan cities. We excluded 148 participants who had been vaccinated for COVID-19. A total of 1735 participants were included in this study. The majority of the participants were 18-39 years old (1285/1735, 74.06%) and identified themselves as male (1638/1735, 94.41%; Table 1). In terms of relationship status, education, and employment status, 67.44% (1170/1735) of participants were currently single, 62.25% (1080/1735) had received a college education or higher, and 69.91% (1213/1735) had a full-time job. Only 77.22% (1339/1735) of the participants had basic health insurance. Moreover, 17.35% (301/1735) of the participants received their HIV diagnosis within 1 year prior, 97.58% (1693/1735) of the participants were on antiretroviral therapy (ART), 70.55% (1224/1735) reported they had an undetectable viral load, and 46.86% (813/1735) reported their CD4 T cell counts were above 500 cells/µL.

**Table 1:** Background characteristics of participants (n=1735).

Sociodemographic characteristics and chronic disease and HIV-related indicators			Results, n (%)
**Age group (years)**			
	18-29	523 (30.14)	
	30-39	762 (43.92)	
	40-49	325 (18.73)	
	≥50	125 (7.20)	
**Gender at birth**			
	Male	1638 (94.41)	
	Female	97 (5.59)	
**Gender identity**			
	Male	1420 (81.84)	
	Female	164 (9.45)	
	Transgender	146 (8.41)	
	Others	5 (0.29)	
**Relationship status**			
	Currently single	1170 (67.44)	
	Cohabited/married with a same-sex partner	236 (13.60)	
	Cohabited/married with an opposite-sex partner	329 (18.96)	
**Highest education level attained**			
	Junior high or below	277 (15.97)	
	Senior high or equivalent	378 (21.79)	
	College and above	1080 (62.25)	
**Employment status**			
	Full-time	1213 (69.91)	
	Part-time/unemployed/retired/students/others	522 (30.09)	
**Monthly personal income (Chinese yuan/US $)**			
	No fixed income	204 (11.76)	
	<1000/154	94 (5.42)	
	1000-2999/154-462	230 (13.26)	
	3000-4999/462-770	501 (28.88)	
	5000-6999/770-1078	338 (19.48)	
	7000-9999/1078-1540)	174 (10.03)	
	≥10,000/1540)	194 (11.18)	
**Type of health insurance**			
	No	197 (11.35)	
	Basic health insurance only	1339 (77.18)	
	Commercial health insurance only	35 (2.02)	
	Both basic and commercial health insurance	157 (9.05)	
	Others	7 (0.40)	
**Study site**			
	Beijing	495 (28.53)	
	Tianjin	320 (18.44)	
	Nanjing	313 (18.04)	
	Hohhot	315 (18.16)	
	Nanning	292 (16.83)	
**Current tobacco use**			
	No	1253 (72.22)	
	Yes	482 (27.78)	
**Current alcohol use**			
	No	1395 (80.40)	
	Yes	340 (19.60)	
**Self-reported BMI (kg/m^2^)**			
	<18.5	155 (8.93)	
	18.5-23.9	1128 (65.01)	
	24.0-27.9	364 (20.98)	
	≥28	88 (5.07)	
**Presence of chronic disease conditions (not including HIV)**			
	No	1157 (66.69)	
	Yes	578 (33.31)	
**Medication use for treating chronic diseases (not including HIV)**			
	No	1639 (94.47)	
	Yes	96 (5.53)	
**History of other vaccinations in the past 3 years**			
	No	1324 (76.31)	
	Yes	411 (23.69)	
**Time since HIV diagnosis (years)**			
	≤1	301 (17.35)	
	2-5	806 (46.46)	
	>5	628 (36.20)	
**On antiretroviral therapy**			
	No	42 (2.42)	
	Yes	1693 (97.58)	
**HIV viral load in the most recent episode of testing (copies/mL)**			
	Undetectable (<50)	1224 (70.55)	
	Detectable (≥50)	197 (11.35)	
	Not sure	314 (18.10)	
**CD4+ T cell count in the most recent episode of testing, cells/mm^3^**			
	>500	813 (46.86)	
	350-499	354 (20.40)	
	200-349	177 (10.20)	
	<200	59 (3.40)	
	Unknown	332 (19.14)	

### Attitudes Toward COVID-19 Vaccines

Regarding the responses to the statement “the likelihood of receiving free COVID-19 vaccination in the future,” 58.4% (1013/1735) of the participants responded that they would accept. Only 2.2% (38/1735) of the participants responded that they would definitely not get vaccinated, 6.7% (116/1735) of the participants said they would probably not get vaccinated, and 32.7% (568/1735) of the participants said they were unsure. In total, 41.6% (722/1735) of participants had vaccine hesitancy (Table S1 in Multimedia Appendix 2).

**Figure 1:**
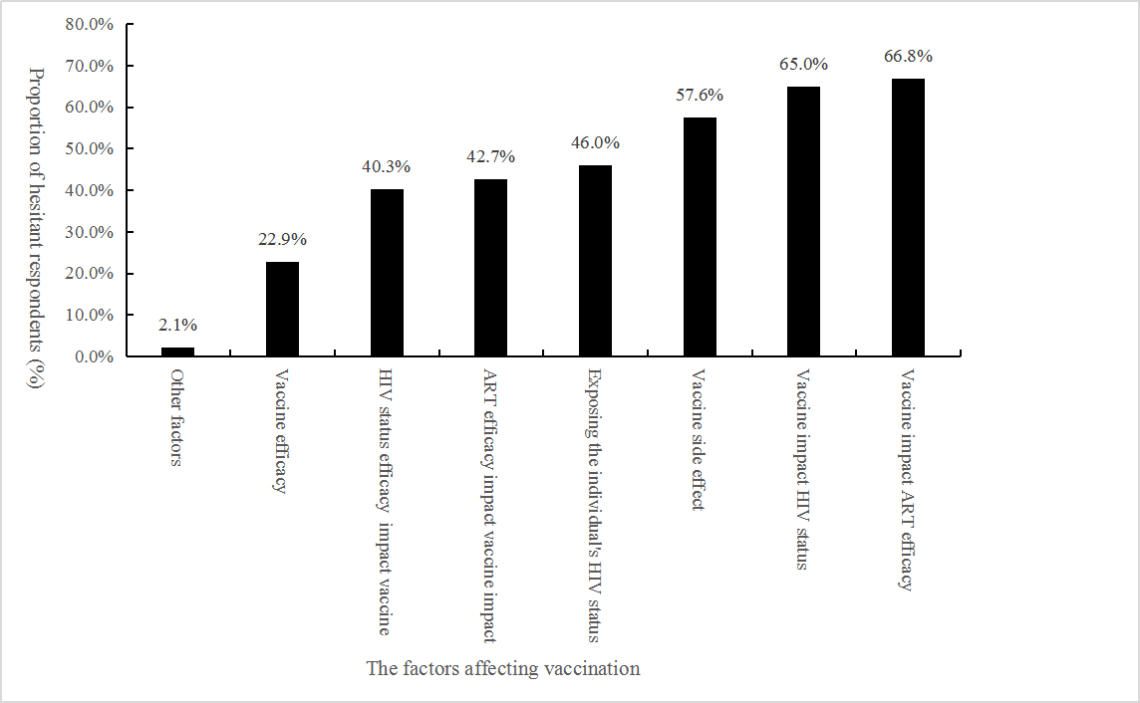
Self-reported reasons of COVID-19 vaccine hesitancy in people living with HIV/AIDS (PLWHA; n=722). ART: antiretroviral therapy.

Among the 722 participants who hesitated to be vaccinated, when they were asked about factors affecting their vaccine willingness, a majority (482/722, 66.8%) of participants demonstrated concerns about a possible influence on ART, and 65% (469/722) had concerns about a possible influence on HIV disease status, that is the HIV disease would progress abnormally, including a rebound of viral load, or a decrease of absolute CD4+ T cell counts after COVID-19 vaccination. Moreover, 57.6% (416/722) of participants had concerns about the possible side effects of the COVID-19 vaccine. Nearly one-half of the participants (332/722, 46%) demonstrated fear of HIV disclosure. Many participants (308/722, 42.7%) had concerns that ART might affect the effectiveness of the vaccine, 40.3% (291/722) of participants worried that their HIV status might affect the effectiveness of the vaccine, and 22.9% (165/722) had concerns about the vaccine effectiveness alone. A small number of participants (15/722, 2.1%) reported other factors that were associated with their vaccine hesitancy (Figure 1).

### Vaccine Hesitancy and Background Characteristics

A 1-way ANOVA was used to assess differences in COVID-19 vaccine hesitancy scores among participants with different demographic characteristics. Compared with the group aged 18-29 years old, participants over 29 years old were more hesitant to get the COVID-19 vaccine (*P*=.009; Table S2 in Multimedia Appendix 2). Participants who had chronic diseases or a chronic disease history were more hesitant than those who did not have (PLWHA without chronic diseases: 2.62 vs PLWHA with chronic diseases or history: 2.42; *P*<.001). PLWHA who did not have other vaccinations in the past 3 years were more hesitant than the ones who did (eg, without other vaccinations: 2.35 vs with other vaccinations: 2.21; *P*=.01).

The significant variables in the univariate analyses were included in the multiple linear regression model. Multiple linear regression analyses identified that the tolerance of independent variables was greater than 0.1, and the variance expansion factor ranged from 1.01 to 1.40. All were less than 10, which indicated there was no multicollinearity and the results of the linear regression model were reliable.

The results of the multivariate linear regression analyses showed that, in general, older age (except for the group aged 40-49 years) was positively associated with COVID-19 vaccine hesitancy. Having received another vaccination in the past 3 years was negatively correlated with COVID-19 vaccine hesitancy (β=–0.07, *P*=.01; Table 2). Having chronic diseases or a chronic disease history was positively correlated with COVID-19 vaccine hesitancy (β=2.77, *P*=.01).

**Table 2:** Multivariate analyses of vaccine hesitancy by background characteristics.

Characteristics		Unstandardized coefficient			Standardized coefficient (β)		*t* value (df)		*P* value		Collinearity statistics	
		B	SE							Tolerance		VIF^a^
Intercept		2.22	0.04	N/A^b^		50.23		<.001		N/A		N/A
**Age (years)**												
	18-29	Ref^c^	Ref	Ref		Ref (3)		Ref		Ref		Ref
	30-39	0.12	0.05	0.06		2.23 (1)		.03		0.71		1.40
	40-49	0.10	0.07	0.04		1.47 (1)		.14		0.73		1.37
	≥50	0.23	0.10	0.06		2.35 (1)		.02		0.83		1.20
Presence of chronic disease conditions		0.14	0.05	0.07		2.77 (1)		.01		0.93		1.07
History of other vaccinations in the past 3 years		–0.15	0.05	–0.07		–2.86 (1)		<.001		1.00		1.01

^a^VIF: variance inflation factor.

^b^N/A: not applicable.

^c^Ref: reference.

### Correlation Matrix

The results showed a negative correlation between perceived benefits and vaccine hesitancy and a positive correlation between perceived risks and vaccine hesitancy (both *P*<.001; Table S3 in Multimedia Appendix 2). Self-efficacy and subjective norms were negatively correlated with vaccine hesitancy (both *P*<.001).

### Measurement Scores

Generally, when the absolute value of the skewness coefficient of an observation variable is <3 and the absolute value of the kurtosis coefficient is <8, the data can be regarded as having a normal distribution. According to the kurtosis results (from –1.29 to 1.45) and skewness (from –1.23 to 0.72), the study data were normally distributed.

The mean self-efficacy score was the lowest of all indicators; in other words, participants’ confidence of COVID-19 vaccination was generally low. The mean perceived risk was the highest. In addition, the concern about HIV disclosure showed the highest mean score among all perceived risks. Moreover, PLWHA would accept the suggestions of family members on COVID-19 vaccination. However, recommendations from a HIV-positive peer and medical professionals had less influence on the acceptance of a COVID-19 vaccine (Table S4 in Multimedia Appendix 2).

### Results of Confirmatory Factor Analysis and SEM

This study hypothesized that perceived risks, perceived benefits, self-efficacy, and subjective norms were associated with COVID-19 vaccine hesitancy; therefore, these 4 factors were included in the SEM to explore their direct or indirect relationships with vaccine hesitancy.

#### Confirmatory Factor Analysis

Confirmatory factor analysis was used to confirm that each latent factor was being measured appropriately. We used the root mean square error of approximation, normed fit index (NFI), incremental fit index (IFI), Tucker-Lewis index (TLI), and comparative fit index (CFI) to assess whether the model was fit appropriately. The values of NFI, IFI, TLI, and CFI were 0.93, 0.94, 0.93, and 0.94, respectively (all >0.90). The results showed that the hypothesized model had an adequate fit (Table S5 in Multimedia Appendix 2).

Table 3 showed that the factor loadings for the items were between 0.52 and 0.92 (above 0.5), and the Cronbach α values were between 0.85 and 0.92. It indicated that this online survey had good reliability. The average variance extracted (AVE) and the construct reliability were above 0.5 and 0.7, respectively, which indicated that the convergent validity and internal consistency of this survey were good. According to the discriminant validity analysis, all square roots of AVE were higher than the correlation values, which indicated a good evaluation (Table 4).

**Table 3:** Results of reliability and convergent validity analyses.

Constructs and items		Measures	Estimate	*P* value		Cronbach α		AVE^a^		CR^b^	
**Perceived benefits**							0.86		0.53		0.87
	PB1	COVID-19 vaccination is effective in improving immune function.	0.65	<.001							
	PB2	COVID-19 vaccination is effective in reducing your risk of SARS-CoV-2 infection.	0.75	<.001							
	PB3	COVID-19 vaccination is effective in reducing mortality caused by COVID-19.	0.86	<.001							
	PB4	COVID-19 vaccination is effective in reducing the severity of COVID-19.	0.81	<.001							
	PB5	COVID-19 vaccination is effective in reducing the risk of spreading.	0.70	<.001							
	PB6	Getting COVID-19 vaccination can make you feel relieved.	0.52	<.001							
**Perceived risks**							0.90		0.58		0.91
	PR1	COVID-19 vaccination has severe side effects.	0.81	<.001							
	PR2	COVID-19 vaccination uptake has a significant negative influence on the effectiveness of ART^c^.	0.76	<.001							
	PR3	COVID-19 vaccination uptake can reduce immunity.	0.70	<.001							
	PR4	You have concerns about the risk of exposing your PLWHA^d^ identity when taking up the COVID-19 vaccine.	0.69	<.001							
	PR5	COVID-19 vaccination uptake can bring trouble/psychological pressure.	0.84	<.001							
	PR6	COVID-19 vaccination uptake may not produce protective antibodies due to HIV infection.	0.80	<.001							
	PR7	The side effects of COVID-19 vaccination are severer for PLWHA than those without HIV infection.	0.70	<.001							
**Self-efficacy**							0.92		0.70		0.92
	SFE1	You will take up the COVID-19 vaccine even if it interrupts your daily routine.	0.79	<.001							
	SFE2	You will get the COVID-19 vaccine even when you do not feel well.	0.81	<.001							
	SFE3	You will get the COVID-19 vaccine even if the side effects would affect your daily activities.	0.92	<.001							
	SFE4	You will get the COVID-19 vaccine even if HIV infection would reduce its effectiveness.	0.84	<.001							
	SFE5	You will get the COVID-19 vaccine even if it reduces the effectiveness of ART.	0.82	<.001							
**Subjective norms**							0.85		0.60		0.86
	SN1	Your family members will support you to get the COVID-19 vaccine.	0.88	<.001							
	SN2	Your HIV-infected friends will support you to get the COVID-19 vaccine.	0.84	<.001							
	SN3	Medical professionals will support you to get the COVID-19 vaccine.	0.74	<.001							
	SN4	CBO^e^ workers will support you to get the COVID-19 vaccine.	0.62	<.001							

^a^AVE: average variance extracted.

^b^CR: construct reliability.

^c^ART: antiretroviral therapy.

^d^PLWHA: person living with HIV/AIDS.

^e^CBO: community-based organization.

**Table 4:** Display discriminant validity analysis.

Constructs	Perceived benefits	Perceived risks	Self-efficacy	Subjective norms
Perceived benefits	0.53	0.32	0.34	0.19
Perceived risks	0.32	0.58	–0.16	–0.19
Self-efficacy	0.34	–0.16	0.70	0.41
Subjective norms	0.19	–0.19	0.41	0.60
The square root of AVE^a^	0.72	0.76	0.84	0.78

^a^AVE: average variance extracted.

#### Structural Equation Modeling

**Figure 2:**
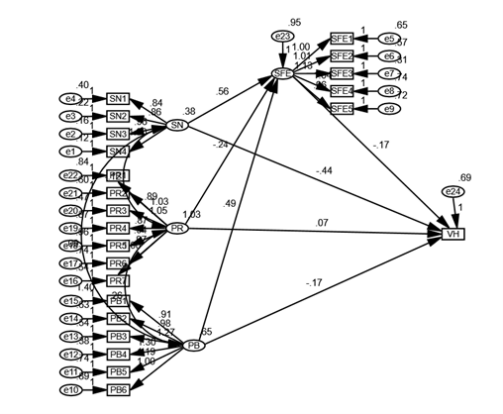
Structural equation modeling results on relationships of perceived benefits (PB), perceived risks (PR), subjective norms (SN), self-efficacy (SFE), and COVID-19 vaccine hesitancy (VH).

Table 5 shows that the results supported hypothesis 1 (H1) to hypothesis 7 (H7). In other words, respondents who had higher perceived benefits, lower perceived risks, higher self-efficacy, and more support from social networks were more willing to receive the COVID-19 vaccine or were less hesitant to be vaccinated against COVID-19. Perceived benefits, perceived risks, and subjective norms yielded significant direct effects on self-efficacy (β=0.35; β=–0.25; β=0.30, respectively; all *P*<.001). The relationship between perceived benefits and vaccine hesitancy was partially mediated by self-efficacy (β=0.03, *P*<.001). The relationship between perceived risks and vaccine hesitancy also was partially mediated by self-efficacy (β=0.08, *P*<.001). Similarly, the relationship between subjective norms and vaccine hesitancy was partially mediated by self-efficacy (β=–0.29, *P*<.001). Furthermore, there were direct relationships between perceived benefits, perceived risks, and subjective norms and COVID-19 vaccine hesitancy (β=–0.15; β=–0.08; β=–0.29; β=–0.20, respectively; all *P*<.001). SEM results are visualized in Figure 2.

**Table 5:** Estimation results of the COVID-19 vaccine hesitancy model.

Hypothesis	Unstandardized path coefficient	Standardized path coefficient	SE	CR^a^	*P* value	Support
H1:PB^b^-VH^c^	–0.17	–0.15	0.03	–5.33	<.001	Yes
H2:PR^d^-VH	0.07	0.08	0.02	2.96	<.001	Yes
H3:SN^e^-VH	–0.44	–0.29	0.04	–11.00	.003	Yes
H4:SFE^f^-VH	–0.17	–0.20	0.02	–7.37	<.001	Yes
H5:PB-SFE	0.49	0.35	0.00	12.20	<.001	Yes
H6:PR-SFE	–0.24	–0.22	0.03	–8.21	<.001	Yes
H7:SN-SFE	0.56	0.30	0.05	11.72	<.001	Yes

^a^CR: critical ratio.

^b^PB: perceived benefits.

^c^VH: vaccine hesitancy.

^d^PR: perceived risk.

^e^SN: subjective norms.

^f^SFE: self-efficacy.

## Discussion

### Principal Findings

This study utilized SEM to investigate relationships among perceived benefits, perceived risks, self-efficacy, subjective norms, and COVID-19 vaccine hesitancy. The study found a high COVID-19 vaccine hesitancy rate among PLWHA in China. Factors associated with vaccine hesitancy were age, a history of chronic diseases, and other vaccinations in the past 3 years. In addition, confidence in COVID-19 vaccination showed the lowest mean of all measured indicators, while perceived risks had the highest mean score. People were highly concerned about possible HIV disclosure during the COVID-19 vaccination. The findings of this study provided valuable information on designing a COVID-19 vaccination campaign addressing possible barriers and improving COVID-19 acceptance among PLWHA.

In this study, 41.61% (722/1735) of participants reported COVID-19 vaccine hesitancy. The rate was higher than the result of 16.4% generated by a previous nationwide online survey in China [33]. Although the updated Chinese guideline included PLWHA for COVID-19 vaccination, PLWHA might have a higher vaccine hesitancy rate than the general population because of concerns about HIV disclosure, interactions with ART and HIV disease, side effects, and others. Moreover, the vaccine hesitancy rate was higher than that of PLWHA in other nations and regions. Various factors might contribute to the difference, such as sociocultural factors, national policy and guidance, and types of vaccines.

This study found that vaccine hesitancy was associated with age, and the relationship showed an inverted U-shaped curve. Except for the group aged 40-49 years, older participants showed higher vaccine hesitancy than the younger group. This finding was consistent with a recent French study [34]. Moreover, we found other vaccinations in the past 3 years and a history of chronic diseases were significant predictors of COVID-19 vaccine hesitancy. PLWHA who did not have other vaccinations in the past 3 years and had a history of chronic diseases were more hesitant be vaccinated against COVID-19. The findings could help promote COVID-19 vaccination among PLWHA. More detailed guidelines on COVID-19 vaccination for people with chronic diseases could be widely disseminated to the public and health care providers. PLWHA and HIV doctors must work on managing chronic diseases and eliminating concerns on COVID-19 vaccination.

We found perceived benefits, perceived risks, and subjective norms yielded significant direct effects on self-efficacy and COVID-19 vaccine hesitancy. The relationships between perceived benefits, perceived risks, subjective norms, and vaccine hesitancy were partially mediated by self-efficacy. The SEM results showed that the higher the perceived benefits, the higher the self-efficacy and the lower the degree of hesitation. Therefore, in order to reduce vaccine hesitation in PLWHA, an education campaign could be developed to provide evidence of the safety and effectiveness of the vaccine, highlighting the latest COVID-19 vaccination guidelines for PLWHA, and informing about the benefits of COVID-19 vaccination from both population and individual perspectives. Previous studies also have highlighted that the safety and efficacy of the COVID-19 vaccine were associated with individuals’ vaccine hesitancy [35,36].

Perceived risks included participants’ perceptions on vaccine safety and the fear of HIV disclosure. The SEM results showed that the higher the perceived risks, the lower the self-efficacy and the higher the degree of hesitation. Moreover, the fear of HIV disclosure during COVID-19 vaccination was a major concern. HIV stigma exists, and people might hesitate to disclose their HIV status when they receive a COVID-19 vaccine. Unintentional HIV disclosure and related stigma might aggravate their psychological burden [8,37,38]. Some strategies could be proposed to address COVID-19 vaccine hesitancy; for example, HIV clinics could collaborate with COVID-19 vaccination sites to provide COVID-19 vaccines to PLWHA. Health care providers at COVID-19 vaccination sites could underline and inform people about a protocol while protecting individuals’ information and privacy.

Subjective norms included the support of family members, HIV-infected friends, medical professionals, and CBO workers. The SEM analysis results showed that, with a higher score for subjective norms, the higher the self-efficacy and the lower the degree of hesitation. PLWHA would prefer to accept suggestions regarding COVID-19 vaccination from the support of their family members. On the other hand, the support of an HIV-positive person and medical professionals showed less influence on PLWHA’s decision making. It showed that PLWHA need the strength of their families. COVID-19 vaccination programs based on PLWHA families could be implemented to improve self-efficacy and reduce vaccine hesitancy in PLWHA through family support and mobilization. Although professional medical providers were one of the most trusted groups that could influence vaccine decision making [39], PLWHA could distrust medical staff because of HIV-related stigma and other reasons [40].

This study had limitations. First, this was a cross-sectional study, so no causality was established. Second, this survey was conducted in PLWHA from 5 large Chinese cities; therefore, the results may not be generalizable to PLWHA in China as COVID-19 vaccine availability, COVID-19 vaccine education, and regional policies and programs might be different among cities and regions. Third, because most of the reported PLWHAs in the 5 selected cities were male, the participants were also majority male. This may influence medical hesitancy, as women are more likely to access medical care. Fourth, because policies and guidelines related to the COVID-19 vaccine have been changing frequently, people’s attitudes about COVID-19 vaccination may vary. Therefore, the findings were sensitive to some factors, such as political and vaccine-related circumstances. Fifth, most measurements in this study were self-constructed and adopted from existing measurements in the general population. The internal validity of these scales was acceptable. However, external validation data were unavailable. Finally, this study did not use random sampling based on the sampling framework, which cannot represent the current situation regarding the vaccination willingness of the entire PLWHA population in China. The extrapolation of the research results needs to be cautious.

### Conclusions

COVID-19 vaccine hesitancy was high among PLWHA in China. To reduce vaccine hesitation and increase vaccine coverage in PLWHA, social sectors, health facilities, and local communities must work on joint efforts and collaborations to implement strategies and programs that increase COVID-19 vaccination efficacy and eliminate barriers.

## Appendix

Multimedia Appendix 1 The process of specific recruitment and data collection.

Multimedia Appendix 2 Supplementary tables.

## Abbreviations

ART: antiretroviral therapy
AVE: average variance extracted
CBO: community-based organization
CFI: comparative fit index
deff: design effect
IFI: incremental fit index
NFI: normed fit index
PLWHA: people living with HIV/AIDS
SEM: structural equation modeling
TLI: Tucker-Lewis index
