# Correction: Reasons for COVID-19 Vaccine Hesitancy Among Chinese People Living With HIV/AIDS: Structural Equation Modeling Analysis

**DOI:** 10.2196/40910

**Published:** 2022-07-29

**Authors:** Yan Yao, Ruiyu Chai, Jianzhou Yang, Xiangjun Zhang, Xiaojie Huang, Maohe Yu, Geng-feng Fu, Guanghua Lan, Ying Qiao, Qidi Zhou, Shuyue Li, Junjie Xu

**Affiliations:** 1 Department of Epidemiology and Biostatistics, School of Public Health Jilin University Changchun China; 2 Department of Preventive Medicine Changzhi Medical College Changzhi China; 3 Department of Public Health University of Tennessee Knoxville, TN United States; 4 Beijing Youan Hospital Capital Medical University Beijing China; 5 Tianjin Centers for Disease Control and Prevention Tianjin China; 6 Jiangsu Provincial Center for Disease Control and Prevention Nanjing China; 7 Guangxi Center for Disease Prevention and Control Nanning China; 8 The Second Hospital of Huhhot Huhhot China; 9 Department of Emergency Medicine, Peking University Shenzhen Hospital Peking University Shenzhen China; 10 Changchun Maternity Hospital Changchun China; 11 Clinical Research Academy, Peking University Shenzhen Hospital Peking University Shenzhen China

In “Reasons for COVID-19 Vaccine Hesitancy Among Chinese People Living With HIV/AIDS: Structural Equation Modeling Analysis” (JMIR Public Health Surveill 2022;8(6):e33995) the authors made the following updates:

1. In the originally published article, author Yan Yao was inadvertently listed as the eleventh author. In the corrected version, author Yan Yao is listed as the first author.

2. In the originally published article, the note of equal contribution was attributed to three authors: Ruiyu Chai, Jianzhou Yang, and Xiangjun Zhang. In the corrected version, the equal contribution note is attributed to authors Yan Yao and Ruiyu Chai.

3. Accordingly, the corrected order of authors is as follows:

Yan Yao^1*^, PhD; Ruiyu Chai^1*^, MM; Jianzhou Yang^2^, PhD; Xiangjun Zhang^3^, PhD; Xiaojie Huang^4^, PhD; Maohe Yu^5^, PhD; Geng-feng Fu^6^, PhD; Guanghua Lan^7^, PhD; Ying Qiao^8^, MM; Qidi Zhou^9^, PhD; Shuyue Li^10^, MM; Junjie Xu^11^, PhD

^*^these authors contributed equally.

4. In the originally published article, author Yan Yao was erroneously listed as the corresponding author. The corresponding authorship is now correctly attributed to Junjie Xu.

5. Accordingly, the address and contact details of the corresponding author have been changed as follows:

Junjie Xu, Phd

Clinical Research Academy

Peking University Shenzhen Hospital

Peking University

1120, Lianhua Road, Futian District

Shenzhen, 518036, China

Phone: 86 755 83923333

Email: xjjcmu@163.com.

6. In the originally published article, the *Authors' Contributions* section was as follows:

RC, YY, and JX conceptualized the study and wrote, reviewed, and edited the manuscript. RC, XH, XZ, SL, and YY designed the methodology. JY, XH, MY, GF, GL, JY, and QZ curated the data. RC, JY, and YY performed the formal analysis. JY, XH, MY, GF, and GL performed project administration. XH and JY obtained the resources. QZ, SL,YY, and JX supervised the study. RC, JY, and XZ contributed equally as first authors. QZ, SL, YY, and JX contributed equally as corresponding authors. All authors have read and approved the final manuscript as submitted.

The corrected *Authors' Contributions* is as follows:

RC, YY, and JX conceptualized the study and wrote, reviewed, and edited the manuscript. RC, XH, XZ, SL, and YY designed the methodology. JY, XH, MY, GF, GL, QY, and QZ curated the data. RC, JY, and YY performed the formal analysis. JY, XH, MY, GF, and GL performed project administration. XH and JY obtained the resources. QZ, SL,YY, and JX supervised the study. YY and RC contributed equally as first authors. JY, XZ, SL and JX contributed equally as corresponding authors. All authors have read and approved the final manuscript as submitted.

The correction will appear in the online version of the paper on the JMIR Publications website on July 29, 2022 together with the publication of this correction notice. Because this was made after submission to PubMed, PubMed Central, and other full-text repositories, the corrected article has also been resubmitted to those repositories.

