# Supplementary material for: Reasons for COVID-19 Vaccine Hesitancy Among Chinese People Living With HIV/AIDS: Structural Equation Modeling Analysis
Source: JMIR Public Health Surveill. 2022 Jun 30;8(6):e33995. doi: 10.2196/33995 (PMC9255267; doi:10.2196/33995)
Supplement: Multimedia Appendix 1 [file publichealth_v8i6e33995_app1.docx]

The process of specific recruitment and data collection

We recruited participants mainly through CBO in five study sites. CBO provides services mainly to the marginalized populations (e.g., PLWHA and HIV high-risk populations) and has been cooperating closely with HIV clinical service providers. In China, HIV outreach services to PLWHA have been transferred from government agencies to CBO. At present, CBO is the primary provider of these routine tasks. What’s more, a large portion of PLWHA is followed up by the CBO. WeChat is the most common live-chat application used by CBO to connect with PLWHA clients. It provides convenience for CBO and PLWHA clients.

First, we provided training for CBO staff who were responsible for communicating with PLWHA in their routine service. Then, the CBO staff posted the study information in the WeChat groups involving PLWHA clients and invited eligible PLWHA to participate. In addition, in order to avoid some participants ignore information, the CBO staff also sent out reminders in the WeChat group.

The CBO staff and prospective participants were asked to keep the study information confidential and not to disclose and disseminate it to anyone outside the PLWHA WeChat groups. If PLWHA were interested in this study, they could contact the CBO staff by private WeChat messages or telephone calls. CBO staff screened prospective participants' eligibility, introduced the study purpose and procedures, answered questions, and explained the confidentiality of study information. Participation in this study was voluntary, and participants could refuse to answer any of the questions and withdraw from the study at any time without any consequences. Participants signed an electronic consent form sent via WeChat message. A link to access an online self-administered questionnaire was sent to the consented participants.

The questionnaire survey was carried out through Golden Data, a commonly used, encrypted web-based survey platform in China. Each individual WeChat account was allowed to access the online questionnaire only once to avoid duplicate responses. The Golden Data tool performed a completeness check before the questionnaire was submitted. Participants could review and change their responses when they completed the questionnaire. Each of participants took about 13-15 minutes to complete this survey. An electronic coupon with a value of 20 Chinese yuan (3.1 US dollars) was sent to the participant upon completion. A unique I.D. was assigned to each participant, which was to delink the study database from personal identifying data. The database we collected was protected by a password and only be accessed by designated research team members.Signed electronic consent forms were kept separately from the empirical data and stored in a password-protected computer or a locked cabinet in the same locked office.
