# Supplementary material for: Reasons for COVID-19 Vaccine Hesitancy Among Chinese People Living With HIV/AIDS: Structural Equation Modeling Analysis
Source: JMIR Public Health Surveill. 2022 Jun 30;8(6):e33995. doi: 10.2196/33995 (PMC9255267; doi:10.2196/33995)
Supplement: Multimedia Appendix 2 [file publichealth_v8i6e33995_app2.docx]

Table S1. Attitudes about COVID-19 Vaccine.

|  |  | N | % |
| --- | --- | --- | --- |
| Accept |  | 1013 | 58.4 |
| Hesitancy |  | 722 | 41.6 |
|  | definitely not | 38 | 2.2 |
|  | probably not | 116 | 6.7 |
|  | unsure | 568 | 32.7 |
| Total |  | 1735 |  |

Table S2. One-way ANOVA analyses of vaccine hesitancy and background characteristics.

|  | Mean | SD | F | *P* |
| --- | --- | --- | --- | --- |
| *Socio-demographics characteristics* |  |  |  |  |
| Age group, years |  |  | 3.89 | .009 |
| 18-29(Reference） | 2.21 | 0.92 |  |  |
| 30-39 | 2.35 | 0.99 |  | .01 |
| 40-49 | 2.34 | 0.91 |  | .04 |
| 50 or above | 2.48 | 0.90 |  | .004 |
| Gender at birth |  |  | 0.70 | .40 |
| Male | 2.31 | 0.95 |  |  |
| Female | 2.39 | 1.06 |  |  |
| Gender identity |  |  | 1.00 | .39 |
| Male | 2.29 | 0.95 |  |  |
| Female | 2.40 | 1.03 |  |  |
| Transgender | 2.39 | 0.90 |  |  |
| Others | 2.40 | 0.55 |  |  |
| Relationship status |  |  | 1.74 | .18 |
| Currently single | 2.31 | 0.95 |  |  |
| Cohabited/married with a same-sex partner | 2.23 | 1.04 |  |  |
| Cohabited/married with an opposite-sex partner | 2.38 | 0.91 |  |  |
| Highest education level attained |  |  | 1.03 | .36 |
| Junior high or below | 2.38 | 0.92 |  |  |
| Senior high or equivalent | 2.27 | 0.90 |  |  |
| College and above | 2.31 | 0.98 |  |  |
| Employment status |  |  | 0.35 | .73 |
| Full-time | 2.32 | 0.96 |  |  |
| Part-time/unemployed/retired/students/others | 2.30 | 0.93 |  |  |
| Monthly personal income, Chinese yuan (U.S. dollar) |  |  | 0.81 | .56 |
| No fixed income | 2.21 | 0.91 |  |  |
| Below 1,000 (154) | 2.44 | 1.11 |  |  |
| 1,000 to 2,999 (154-462) | 2.31 | 0.94 |  |  |
| 3,000 to 4,999 (462-770) | 2.30 | 0.91 |  |  |
| 5,000 to 6,999 (770-1,078) | 2.34 | 0.95 |  |  |
| 7,000 to 9,999 (1,078-1,540) | 2.31 | 1.03 |  |  |
| At least 10,000 (1,540) | 2.36 | 0.98 |  |  |
| Type of health insurance |  |  | 0.57 | .68 |
| No | 2.29 | 0.98 |  |  |
| Basic health insurance only. | 2.32 | 0.95 |  |  |
| Commercial health insurance only. | 2.11 | 0.87 |  |  |
| Both basic and commercial health insurance | 2.34 | 0.98 |  |  |
| Others | 2.57 | 0.98 |  |  |
| Current tobacco use |  |  | 2.33 | .13 |
| No | 2.29 | 0.96 |  |  |
| Yes | 2.37 | 0.94 |  |  |
| Current alcohol use |  |  | 0.84 | .36 |
| No | 2.32 | 0.95 |  |  |
| Yes | 2.27 | 0.96 |  |  |
| Self-reported BMI^a^, kg/m² |  |  | 0.78 | .50 |
| <18.5 | 2.33 | 0.91 |  |  |
| 18.5-23.9 | 2.30 | 0.93 |  |  |
| 24.0-27.9 | 2.37 | 1.06 |  |  |
| ≥28 | 2.23 | 0.85 |  |  |
| *Chronic diseases and HIV related indicators* |  |  |  |  |
| Presence of chronic disease conditions(not including HIV) |  |  | 10.72 | .001 |
| No | 2.26 | 0.95 |  |  |
| Yes | 2.42 | 0.96 |  |  |
| Medication use for treating chronic diseases(not including HIV) |  |  | 2.04 | .15 |
| No | 2.31 | 0.95 |  |  |
| Yes | 2.45 | 1.07 |  |  |
| History of other vaccinations in the past three years |  |  | 6.70 | .01 |
| No | 2.35 | 0.97 |  |  |
| Yes | 2.21 | 0.90 |  |  |
| Time since HIV diagnosis, years |  |  | 0.84 | .43 |
| ≤1 | 2.28 | 0.92 |  |  |
| 2-5 | 2.29 | 0.96 |  |  |
| >5 | 2.35 | 0.96 |  |  |
| On antiretroviral therapy (ART) |  |  | 0.46 | .50 |
| No | 2.21 | 1.03 |  |  |
| Yes | 2.32 | 0.95 |  |  |
| HIV viral load in the most recent episode of testing, copies/ml |  |  | 1.27 | .28 |
| Undetectable (<50) (Reference） | 2.33 | 0.95 |  |  |
| Detectable(≥50) | 2.33 | 1.01 |  |  |
| Not sure | 2.24 | 0.94 |  |  |
| CD4+ T cell count in the most recent episode of testing, cells/mm^3^ |  |  | 1.44 | .22 |
| >500 | 2.35 | 0.95 |  |  |
| 350-499 | 2.26 | 0.92 |  |  |
| 200-349 | 2.22 | 0.95 |  |  |
| <200 | 2.46 | 1.02 |  |  |
| Unknown | 2.29 | 0.98 |  |  |

### Note, ^a^BMI: Body Mass Index.

Table S3. Correlation matrix.

| Variables | Vaccine hesitancy | Perceived benefits | Perceived risks | Self-efficacy | Subjective norms |
| --- | --- | --- | --- | --- | --- |
| Vaccine hesitancy | 1 | -0.26^a^ | 0.11^a^ | -0.37^a^ | -0.42^a^ |
| Perceived benefits | -0.26^a^ | 1 | 0.28^a^ | 0.34^a^ | 0.20^a^ |
| Perceived  risks | 0.11^a^ | 0.28^a^ | 1 | -014^a^ | -0.19^a^ |
| Self-efficacy | -0.37^a^ | 0.34^a^ | -0.14^a^ | 1 | 0.40^a^ |
| Subjective norms | -0.42^a^ | 0.20^a^ | -0.19^a^ | 0.40^a^ | 1 |

^a^*P*<.01.

Table S4. Measurement scores of the participants.

| Construct | Item | Measures | Score | Mean | SD^a^ | Skewness | Kurtosis |
| --- | --- | --- | --- | --- | --- | --- | --- |
| Perceived Benefits |  |  |  | 3.69 | 0.96 | -0.52 | -0.15 |
|  | PB1 | COVID-19 vaccination is effective in improving immune function | 1~5 | 3.20 | 1.39 | -0.22 | -1.11 |
|  | PB2 | COVID-19 vaccination is effective in reducing your risk of SARS-CoV-2 infection | 1~5 | 3.91 | 1.12 | -0.86 | 0.08 |
|  | PB3 | COVID-19 vaccination is effective in reducing mortality caused by COVID-19 | 1~5 | 3.72 | 1.26 | -0.70 | -0.45 |
|  | PB4 | COVID-19 vaccination is effective in reducing the severity of COVID-19 | 1~5 | 3.76 | 1.22 | -0.70 | -0.39 |
|  | PB5 | COVID-19 vaccination is  effective in reducing the risk of spreading | 1~5 | 3.73 | 1.29 | -0.75 | -0.46 |
|  | PB6 | Taking up COVID-19 vaccination can make you feel relieved | 1~5 | 3.81 | 1.24 | -0.79 | -0.32 |
| Perceived Risks |  |  |  | 3.71 | 1.03 | -0.80 | 0.13 |
|  | PR1 | COVID-19 vaccination has severe side effects | 1~5 | 3.33 | 1.29 | -0.30 | -0.86 |
|  | PR2 | COVID-19 vaccination uptake has a significant negative influence on the effectiveness of ART | 1~5 | 3.81 | 1.30 | -0.80 | -0.47 |
|  | PR3 | COVID-19 vaccination uptake can reduce immunity | 1~5 | 3.82 | 1.27 | -0.82 | -0.34 |
|  | PR4 | You have concerns about the risk of exposing your PLWHA identity when taking up the COVID-19 vaccination | 1~5 | 4.08 | 1.29 | -1.23 | 0.33 |
|  | PR5 | COVID-19 vaccination uptake can bring trouble/psychological pressure | 1~5 | 3.41 | 1.37 | -0.40 | -0.98 |
|  | PR6 | COVID-19 vaccination uptake may not produce protective antibodies due to HIV infection | 1~5 | 3.72 | 1.31 | -0.69 | -0.61 |
|  | PR7 | The side effects of COVID-19 vaccination are severer for PLWH than those without HIV infection | 1~5 | 3.82 | 1.25 | -0.78 | -0.41 |
| Self-Efficacy |  |  |  | 2.566 | 1.41 | 0.40 | -1.05 |
|  | SFE1 | You will take up COVID-19 vaccination even if it interrupts your daily routine | 1~5 | 2.87 | 1.40 | 0.07 | -1.19 |
|  | SFE2 | You will take up COVID-19 vaccination even when you do not feel well | 1~5 | 2.43 | 1.38 | 0.54 | -0.93 |
|  | SFE3 | You will take up COVID-19 vaccination even if the side effects would affect your daily activities | 1~5 | 2.51 | 1.41 | 0.45 | -1.06 |
|  | SFE4 | You will take up the COVID-19 vaccine even if HIV infection would reduce its effectiveness | 1~5 | 2.73 | 1.47 | 0.23 | -1.29 |
|  | SFE5 | You will take up the COVID-19 vaccine even if it reduces the effectiveness of ART | 1~5 | 2.26 | 1.39 | 0.72 | -0.78 |
| Subjective Norms |  |  |  | 3.32 | 0.61 | 0.31 | 1.45 |
|  | SN1 | Your family members will support you to take up the COVID-19 vaccine | 1~5 | 3.42 | 0.82 | 0.01 | 0.33 |
|  | SN2 | Your HIV-infected friends will support you to take up the COVID-19 vaccine | 1~5 | 3.19 | 0.71 | 0.23 | 1.42 |
|  | SN3 | Medical professionals will support you to take up the COVID-19 vaccine | 1~5 | 3.33 | 0.72 | 0.19 | 0.92 |
|  | SN4 | CBO workers will support you to take up COVID-19 vaccine | 1~5 | 3.35 | 0.71 | 0.33 | 1.05 |

Note, ^a^SD: Standard Deviation.

Table S5. Confirmatory Factor Analysis of model fit.

| Fitting index | RMSEA^a^ | NFI^b^ | IFI^c^ | TLI^d^ | CFI^e^ |
| --- | --- | --- | --- | --- | --- |
| Estimated Model | 0.06 | 0.93 | 0.94 | 0.93 | 0.94 |
| Acceptable range | ＜0.08 | >0.90 | >0.90 | >0.90 | >0.90 |

Note, ^a^RMSEA: Root Mean Squared Error of Approximation; ^b^NFI:Normed Fit Index; ^c^IFI : Incremental Fit Index; ^d^TLI:Tucker-Lewis Index; ^e^CFI: Comparative Fit Index.
